# Biochemistry Learning in Higher Education: A Systematic Review on Methodologies and Teaching Resources

**DOI:** 10.1002/bmb.70027

**Published:** 2025-11-22

**Authors:** Micaela Jardim e Silva, Mariana Silva Cecilio, Maurícius Selvero Pazinato

**Affiliations:** ^1^ Federal University of Rio Grande do Sul Porto Alegre Brazil

**Keywords:** higher education, systematic review, teaching methodologies, teaching of biochemistry, teaching resources

## Abstract

The teaching of biochemistry in higher education presents several challenges, including the complexity of molecular visualization, the use of technical terminology, and the vast amount of content covered in courses. This systematic review explores current research on biochemistry teaching at the higher education level published between 2012 and 2024, focusing on the teaching resources and methods used. A total of 271 articles selected from the ERIC and SciFinder databases were analyzed, categorizing the teaching resources and methodologies employed, highlighting the main contributions of the works to the teaching of biochemistry. Most of the identified didactic resources involved the use of digital technologies, primarily to assist in spatial visualization of the molecular structures of biomolecules and to improve the understanding of fundamental concepts. Considering teaching methodologies, lecture‐style classes still predominated, although innovative approaches such as the flipped classroom, problem‐based learning, and team‐based learning are increasingly being integrated into biochemistry education. Furthermore, the techniques of virtual and augmented reality, interactive multimedia, and digital simulations are emerging as promising ways to enhance student understanding and engagement. This review gives teachers a comprehensive overview of recently published knowledge concerning biochemistry teaching, providing a foundation for the implementation of innovative and effective educational practices.

## 1 Introduction

Teaching Biochemistry to undergraduate students, regardless of their field of study, often faces significant challenges, because common difficulties reported by students include visualizing molecular structures, the use of technical terminology, and the sheer volume of concepts covered in classes [1, 2, 3], resulting in unsatisfactory performance [4]. In addition, there is growing concern about student motivation and engagement, while the perceived relevance of biochemistry to their future professions may not be immediately obvious [5, 6, 7].

Therefore, the purpose of this article is to investigate current research related to the teaching of biochemistry at the higher education level, presenting the teaching resources and methods that have been used in research studies reported in the literature between 2012 and 2024. The aim is to assist educators by providing a solid basis for innovation in established teaching practices, or the creation of new ones, based on recent research carried out in the classroom.

## 2 Methods

### 2.1 Methodological Choices and Research Questions

This systematic review was carried out in accordance with the principles of qualitative research [8]. We began by establishing our search strategy, which was guided by the following research questions: *What teaching resources have been used in biochemistry classes in higher education?* and *What teaching methods have been used to teach biochemistry in higher education?* Our goal was to identify articles that described teaching methods or teaching resources and their application in classroom settings within higher education. This approach, inductive and exploratory, was intended to allow the literature itself to reveal the most prominent practices and strategies currently being used in biochemistry teaching. To answer these questions, the proposals of Wei et al. [9] were used to define the databases and it was endeavored to meet the guidelines of PRISMA [10]. Furthermore, this study relied exclusively on data extracted from publicly available published articles and did not involve direct interaction with human participants. Therefore, ethical approval from a research ethics committee was not required.

The search for articles was performed using two databases: (1) Education Resources Information Center (ERIC) and (2) SciFinder. This decision was guided by the nature of our research, which intersects both education and biochemistry, and by the relevance and comprehensiveness of these two databases in their respective fields. ERIC is widely recognized as one of the most comprehensive and specialized databases for educational research, while SciFinder is a leading resource in the fields of chemistry and biochemistry. By combining these two sources, we aimed to ensure the retrieval of peer‐reviewed, high‐quality research aligned with our guiding questions.

We acknowledge, however, that this choice imposes a limitation on the scope of the review. Relevant studies indexed in other databases may have been excluded. Furthermore, both databases primarily index articles published in English, which may have led to the exclusion of potentially relevant works written in other languages.

### 2.2 Article Selection Process

The article selection process for this systematic review was carried out in three stages: an exploratory testing of keywords, database search and screening of eligible studies. We initially conducted exploratory searches using a variety of terms and combinations, such as Education, Biochemistry, Teaching method, Active learning, Science Education, Lipids, Proteins, Carbohydrates, Teaching Resources, Educational tools, among others. This preliminary step aimed to identify the most effective search terms capable of retrieving a high number of relevant records specifically related to teaching practices in Biochemistry, while remaining aligned with the guiding questions of the review. This step proved necessary, as using only the term “biochemistry education” returned a very limited number of results, whereas the broader combination “biochemistry” AND “education” generated an excessively high number of results, many of which were unrelated to the teaching of Biochemistry.

Based on this initial exploration, our final search strategy was defined around three main keywords: ‘biochemistry’, ‘active learning’, and ‘teaching method’. These terms were combined using the Boolean operator AND in different ways.

They were selected to capture the intersection between the disciplinary content (Biochemistry) and the pedagogical approach (teaching methods or resources used in Biochemistry education).

In the ERIC database, the search was conducted in the title field and a specific time period, using descriptors provided by the database (*pubyearmin* and *pubyearmax*). In the SciFinder database, the process differed slightly, as it allows the application of filters for publication period and language, which were both selected during the search. In each database, the following search terms were used: (1) “biochemistry” AND “teaching method”; (2) “biochemistry” AND “active learning”.

The criteria for selecting the articles were: (i) original research studies published in peer‐reviewed journals; (ii) publication date between 2012 and 2024; (iii) language of publication in English, Portuguese, or Spanish; (iv) the articles must present quantitative or qualitative evidence derived from a research methodology. The exclusion criteria were: (i) articles not involving classroom applications or empirical studies; (ii) theoretical essays, editorials, chapter books, reviews, or opinion pieces; (iii) studies not focused on Biochemistry teaching; (iv) duplicated articles across databases.

Between July 2023 and May 2025 two researchers conducted the full analysis of the selected articles. An analytical matrix was developed and continuously refined to guide the synthesis of data, based on the research questions. The matrix included key variables such as study type, teaching method or resource, educational level, and main outcomes. Each article was initially analyzed by one researcher and subsequently reviewed by the second, ensuring consistency of categorization. In cases where the classification was unclear, especially when articles presented overlapping approaches, such as combining methods or integrating digital tools with teaching methods, discussions were held to reach consensus. The categorization of each article followed a principle of mutual exclusivity: each study was assigned to only one category and subcategory, based on the authors' stated emphasis and definitions. This decision aimed to ensure analytical clarity and prevent overrepresentation of individual studies.

**FIGURE 1:**
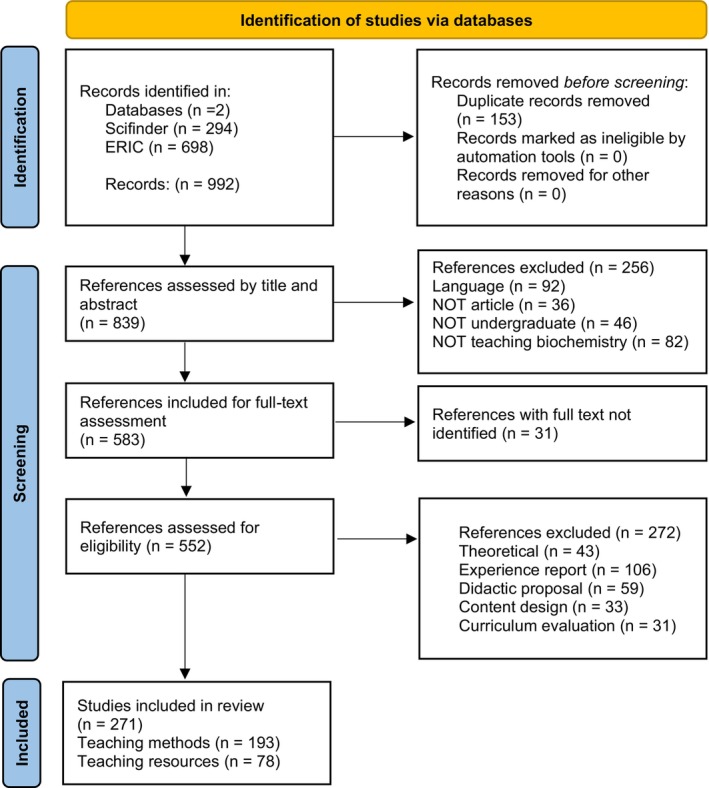
Scheme based on the PRISMA guidelines, showing the stages for selection of the articles used in this study.

A structured overview of the article selection process is illustrated in Figure 1, following the PRISMA guidelines. The diagram presents the number of articles identified, screened, and included in the final analysis, along with reasons for exclusion at each stage.

In the identification stage, a total of 992 articles were found, of which 294 were in the Scifinder database and 698 were in the ERIC database. Of these, 256 were excluded because they did not meet the established criteria. As a result, 583 articles were considered eligible for full‐text reading. Of this total, only 271 articles (Supporting Information) were deemed relevant for answering the questions guiding this review, classified in the categories Teaching Resources (78 articles) and Teaching Methods (193 articles).

## 3 Results and Discussion

**FIGURE 2:**
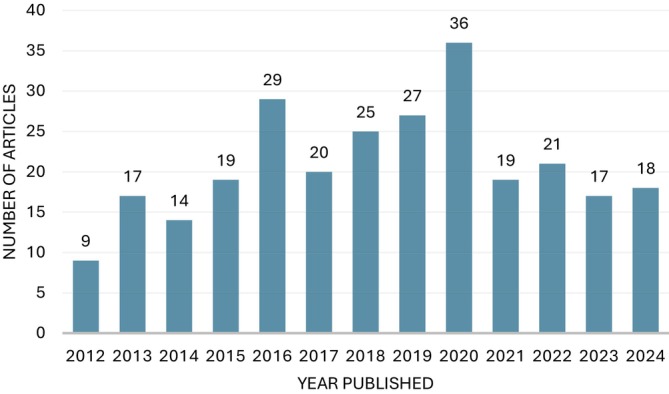
Number of articles published per year.

The 271 selected articles were published in 43 different journals, highlighting Biochemistry and Molecular Biology Education (BAMBED), with 168 papers (62%), consistent with its tradition and importance in disseminating research related to the area of biochemistry education. The studies were carried out in 36 different countries, especially the United States of America (45.4%), China (9.6%), India (6.6%), Brazil (4.4%), and Canada (4.4%). Figure 2 shows the numbers of articles published annually between 2012 and 2024.

### 3.1 What Teaching Resources Have Been Used in Biochemistry Classes in Higher Education?

The reviewed studies revealed the use of a variety of teaching resources. It is important to note that some categories inevitably overlap, as already discussed in the methodology section. In such cases, the classification followed the focus stated by the authors to avoid the overrepresentation of articles that employ more than one teaching resource. Table 1 shows five subcategories that reflect the different teaching resources identified in the articles.

**TABLE 1:** Description of the subcategories of teaching resources.

Subcategory	Number of articles	Description
Digital Tools	25	Use of digital tools such as software and databases, applied in the classroom or remotely.
Models and Games	24	Use of molecular models and/or analog games in the classroom.
Online Activities	12	Use of online activities based on websites, in the classroom or remotely.
Multimedia	11	Use of videos, music, illustrations, and animations, in the classroom or remotely.
Immersive Technology	6	Use of resources based on virtual reality (VR) and augmented reality (AR), in the classroom or remotely.

#### 3.1.1 Digital Tools

The use of 3D visualization and simulation software emerged as the most prominent teaching resource in this subcategory, being found in 13 articles [11, 12, 13, 14, 15, 16, 17, 18, 19, 20, 21, 22, 23]. These tools enable students to visually explore molecules in three dimensions.

The implementation of digital platforms and applications has shown benefits in terms of increasing the engagement of students and improving the effectiveness of their learning processes. The work by McQueen et al. [24] illustrates this effect achieved using PeerWise, a platform that allows students to create, answer, and discuss multiple‐choice questions. Research by Wang et al. [25], Shu et al. [26] and Ding [27] explored how online platforms can provide educational resources and enhance direct interaction between students and teachers. By enabling instant feedback, such platforms can significantly contribute to the creation of a more collaborative educational environment. The use of a quiz and revision application represents another differentiated approach to assessment and knowledge reinforcement [28].

The introduction of virtual laboratories [29, 30, 31, 32, 33] and a remote experiment [34] shows the possibility of providing a virtual hands‐on experience, allowing students to carry out experiments remotely. Rossi et al. [35] investigated the implementation of active teaching tools in an online science course during the COVID‐19 pandemic. The results corroborated those of the other studies presented in this subcategory, suggesting that the use of active learning tools in online environments can be effective in encouraging student engagement.

#### 3.1.2 Models and Games

Molecular models are educational tools that make it possible to refine students' mental models of molecular structures and processes. In the reported works, these models were used in studies of enzymes [36], gene expression [37], electrostatic interactions [38], DNA and RNA [37, 39, 40, 41], proteins [42, 43, 44], and allosteric regulation [43]. These investigations included an innovative approach proposed by Srivastava [39], who described the benefits of dissecting models, as opposed to their traditional construction (representing the structures of molecules). This perspective brings a new dimension to learning about molecular structure, promoting a deeper and more practical understanding of fundamental concepts.

Other types of models have been described in studies exploring the use of alternative materials, such as paper origami [45], simple objects [46], Pop‐It Beads [47], and LEGO [48]. Another way of using models as teaching tools is by means of simple graphical representations, as described by Bonafe et al. [49] and Arya and Kumar [50].

The use of classical games as accessible resources has been reported to enrich the learning process. This includes card and board games [51, 52, 53, 54], which addressed topics such as amino acid recognition, vitamin metabolism, and structure–function relationships in biochemistry, aiming to enhance conceptual understanding and spatial visualization. Additionally, a hangman's game [55], with a focus on memorizing the different amino acids and intended to encourage participation of the students, and self‐made crossword puzzles [56], in an approach that challenges the lexical knowledge of students.

Among the games that deviate from the classics, the interactive game DNA Re‐EvolutioN [57] proved to be effective in teaching genetics concepts, including DNA structure, while improvements in student performance and engagement were achieved using iM‐tool [58], in which metabolic pathways are represented. The tool led to a significant increase in student test scores, with average scores rising from 46.42% to 62.00%, a robust improvement of 15.58% as well as a 17% increase in course pass rates. Elsewhere, the Chromosome Connections Kit [59] helped students to understand the process of meiosis and associated concepts, combining an interactive introductory video and a model‐based activity.

#### 3.1.3 Online Activities

A notable feature of the research in this subcategory is the versatility of online features to promote interactive learning [60, 61, 62, 63, 64, 65, 66, 67, 68, 69, 70, 71]. Examples are the use of emojis as visual representations of neurotransmitters in the game “N.A.M.E.” FUN! [60], in which students interact with the content by dragging objects to their correct locations or by answering specific questions within a metabolic pathway [61], as well as the introduction of the Mobile‐Based Cooperative Learning platform [62]. Additionally, gamification has been explored to enhance scientific reading skills, as seen in the game “Discovering a Glycoprotein: The Case of the H,K‐ATPase”, developed using Genially to engage students in research‐based learning [68]. Online approaches have also been applied to foster research skills, such as the Wintersession Research Week, a remote initiative that introduced independent research in STEM, strengthening their scientific identity and confidence [70]. Similarly, an interdisciplinary course in Computer‐Aided Drug Discovery (CADD) successfully combined virtual research experiences in bioinformatics and molecular modeling to engage students in real‐world scientific challenges [71]. These tools illustrate how technology can be used to create learning experiences that encourage student participation in a collaborative way.

Also in this subcategory, studies have shown how the use of online resources can improve the preparedness of students for practical biochemistry classes, highlighting the importance of structured prior learning [63, 64]. Also noteworthy is a study demonstrating that the guided use of e‐journals can increase student engagement in critical reading and application of knowledge [65], as well as two articles reporting the successful adaptation of the use of iPads for remote biochemistry teaching during the COVID‐19 pandemic [66, 69].

#### 3.1.4 Multimedia

Various articles have reported on the use of videos [72, 73, 74], music [75], illustrations [76], and animations [77, 78, 79, 80, 81] to teach different biochemistry topics. Animations can be highlighted, especially when used to clarify metabolic pathways, given the difficulty students have in understanding their interrelationships [77, 79, 80, 81]. To this end, Lee et al. [79, 80] developed interactive self‐learning tools, including *AG City* and *Metabolism Metro*, which use a subway map as a conceptual model to illustrate metabolic pathways and their integration. Metabolism Metro expands this approach by incorporating pre‐class animations, revision flashcards, and self‐assessment quizzes, following an Agile adaptation of the ADDIE instructional design for continuous improvement. Long et al. [77] developed a 3D animation that introduces the fundamental concepts of glucose and fat metabolism for energy production, emphasizing the integration and flows of these metabolic pathways. In the study by Goff et al. [81], it was shown that static images can be effective in teaching metabolic pathways, although the advantages of animations, compared to static images, were highlighted for this specific purpose. Similarly, Costabile [82] demonstrated how a short, interactive simulation significantly improved student understanding, reinforcing the role of animations and simulations in simplifying complex physiological and immunological concepts. The authors emphasized the importance of using different formats of visual representations when teaching complex biochemistry and molecular biology concepts.

#### 3.1.5 Immersive Technology

The use of resources based on virtual reality (VR) and augmented reality (AR) represents an innovative and emerging approach in the educational field. Four studies were identified that used VR [83, 84, 85, 86, 87, 88] to interact with 3D molecules [83], to teach about DNA [84], to address concepts related to photosynthesis and electron transport [85], and to learn about enzymatic catalysis [86]. This tool provided satisfactory results in terms of understanding complex molecular behavior and improving conceptual learning.

In the case of AR, Peterson et al. [87] used Microsoft HoloLens in the teaching of the three‐dimensional molecular structures of proteins and DNA. According to the research participants, this resource improved the understanding and visualization of these structures. Despite the significant advantages, it is important to note that glasses suitable for implementing AR or VR activities are expensive, which may limit their use in many universities. Furthermore, implementing these technologies in the classroom requires specific technical skills, including familiarity with AR/VR software and hardware, which may pose a challenge for instructors without this expertise. Additionally, the use of these technologies can cause discomfort in some users, such as vertigo, which is a concern to consider in implementation. However, the article by Argüello and Dempski [88] described an affordable solution for visualizing proteins using mobile devices, promoting inclusion and student engagement.

### 3.2 What Teaching Methods Have Been Used to Teach Biochemistry in Higher Education?

The selected articles present a variety of teaching methods and their combinations. As with the categorization of resources, some methodological approaches inevitably share common features and may overlap. To address this, the classification was guided by the main emphasis indicated by the authors, thus preventing the same study from being counted more than once. Table 2 lists 10 subcategories that encompass these various methodologies discussed in recent studies reported in the literature.

**TABLE 2:** Description of the teaching method subcategories.

Subcategory	Number of articles	Description
Experimental Activities	50	Focus is on the use of experimental activities as a teaching methodology, involving either individual experiments or a series of experiments.
Other Methods	25	Teaching methods not covered by the previous subcategories, encompassing a variety of alternative instructional approaches.
Inquiry‐Based Learning	22	Applied inquiry‐based learning, in which students work collaboratively with materials provided by the teacher, answering questions and carrying out activities and/or experiments.
Lecture‐Based Teaching Variations	21	Application of innovative strategies and modifications to traditional lecture‐based teaching.
Problem‐Based Learning	21	An instructional approach where students are challenged to solve real‐world problems employing case studies or scenarios.
Flipped Classroom	17	A teaching method that reverses the traditional classroom model by having students learn material before class and then apply it in class.
Literature‐Based Learning	12	An instructional approach where teaching is centered around reading and discussing scientific literature.
Project‐Based	10	An instructional approach where students actively engage in hands‐on projects to explore and solve real‐world problems and challenges.
Team‐Based Learning	10	A structured instructional method that fosters active learning by means of collaborative group work and individual accountability.
Educational Product	5	Development of an educational product as part of the learning process.

#### 3.2.1 Experimental Activities

Experimental activities are often used to demonstrate or verify concepts worked on in the classroom, with a currently recommended approach being to combine them with investigative approaches [89]. A good example is the Course‐Based Undergraduate Research Experience (CURE) method, which consists of an educational model that integrates research experience into undergraduate courses. In this review, 35 studies were found that proposed experiments for teaching different biochemistry contents [90, 91, 92, 93, 94, 95, 96, 97, 98, 99, 100, 101, 102, 103, 104, 105, 106, 107, 108, 109, 110, 111, 112, 113, 114, 115, 116, 117, 118, 119, 120, 121, 122, 123, 124, 125], while 13 studies focused on implementing the CURE approach [126, 127, 128, 129, 130, 131, 132, 133, 134, 135, 136, 137, 138]. Aspects of these studies that could be highlighted included the use of experimental activities to teach the structure–function of proteins and enzymes [90, 91, 92, 93, 94, 95, 98, 100, 115], as well as the contextualization of biochemistry using the theme of food safety [96, 97, 101, 102].

Some studies have proposed the use of advanced instrumental techniques, such as nuclear magnetic resonance (NMR), in the teaching of proteins [93] and phospholipids [104], as well as spectroscopic techniques [105]. Experiments have also concerned CRISPR [106, 107, 108] and PCR [109, 110] techniques, the antimicrobial properties of essential oils [111], the study of gene expression [112], the identification of lymphocytes [113], protein colorimetry techniques [114], and the biochemistry of urine in the diagnosis of diseases [116]. Studies concerning laboratory experiments focusing on “Predict, Observe, Explain” (POE) [117] and its variations [118, 119, 120] have also shown benefits for student learning, compared to merely demonstrative experiments. This was shown in a study on optimizing laboratory practices for teaching biochemistry, from the perspectives of teachers and students [121]. The CURE methodology has been shown to considerably improve the ability of students to understand complex scientific concepts and the processes involved [126, 127, 128, 129, 130, 131, 132, 133, 134, 135, 136, 137, 138, 139], and has been accompanied by increases in confidence and the ability to carry out research independently [126, 136]. In addition, the development of specific practical skills has been reported, such as cell culture [129, 133], protein purification [126, 133], and gene expression analysis [128, 134, 136].

#### 3.2.2 Other Methods

Peer‐to‐peer learning is an active methodology that encourages communication between students and can promote more productive study sessions. Cansever et al. [140] reported good adherence of the students, some of whom also showed greater interest in their academic careers after completing the proposed activities, while Higgins et al. [141]. Analyzed the use of PeerWise and found a positive correlation between student engagement and academic performance.

Using some of the fundamentals of the peer learning method, Wiles [142] applied a methodology based on the analysis of figures as a way of developing skills related to the interpretation of graphs, schematics, and diagrams. Hancock et al. [143] described the process of implementing a methodology based on student participation in preparing and solving multiple‐choice questions. Simply solving multiple‐choice questions is commonly associated with shallow learning, but when combined with the PeerWise platform, the authors showed that student performance was proportional to their engagement with the platform. Horn and Hernick [144] also explored question‐solving in the learning of concepts related to lipids.

Along these lines, Bobby et al. [145] proposed a variation of the PBL methodology, investigating the effects of an individual revision activity in which the students were given information concerning bioenergetics and biological oxidation, and had to assess it as correct or incorrect, justifying their responses. Other studies investigated the combined use of various active methodologies mentioned in this review, in certain classes, with good results obtained, comparing the performance outcomes with those achieved for traditionally taught classes [146, 147, 148, 149].

In another report, a comparison was made between two cooperative methodologies for learning about hormones, namely team tournament and a jigsaw puzzle, highlighting the greater benefits of the latter methodology, in terms of understanding the content [150].

Goeden et al. [151] introduced a community‐based inquiry methodology, which encouraged students to carry out their own laboratory investigations, addressing real needs in their communities. Da et al. [152] found that the use of clinical assessment exercises, in an integrated‐based learning context, led to significant improvements in student performance in physical examinations, organizational effectiveness, and competence, as well as in the application of basic knowledge.

Tian et al. [153] reported the application of the Ausubel theory of cognitive assimilation to the teaching of biochemistry. The authors highlighted the importance of helping students overcome difficulties in conceptual understanding by means of the development of cognitive skills and advanced organizers. Ouyang et al. [154] presented a combination of strategies, such as the use of presentation‐assimilation‐discussion (PAD) pedagogy, analysis of real data, and intensive reading of scientific articles, to teach genomics concepts. The work of La Frano et al. [155] described the participation of undergraduate students in a controlled feeding study with metabolomics analysis, which was a practical approach that improved their understanding of metabolism. This research‐based learning experience highlighted the importance of the practical application of theoretical knowledge, stimulating the enthusiasm and active participation of the students.

Hybrid and blended learning models have also been explored to enhance student engagement and performance. A study by Sun et al. [156] demonstrated that a hybrid model combining virtual simulations with in‐person lab activities significantly improved students' laboratory skills and theoretical knowledge retention. Similarly, Lu et al. [157] found that a blended teaching approach was more effective than traditional methods in fostering self‐directed learning abilities among medical students. Additionally, Sancassani et al. [158] investigated the impact of a transdisciplinary workshop designed to connect organic chemistry and biochemistry concepts, reinforcing students' understanding through collaborative learning and problem‐solving activities. The results indicated that integrating disciplines in an active learning environment enhances conceptual comprehension and soft skills development.

Two studies were also found that reported teaching methodologies involving the use of games to assist learning. The first aimed to promote the memorization of biochemistry concepts, such as by using mnemonics applied to the Krebs cycle [159], while the second proposed a role‐playing activity to learn about the central themes of molecular biology [160].

Three articles were found that made significant contributions to the integration of academic research concerning innovations in biochemistry teaching. In one study, a bioinformatics course was developed where undergraduate students were integrated into computational genetics research, providing a practical experience of bioinformatics research [161]. Another article reported the effectiveness of the “Writing in Your Own Voice” intervention, which significantly reduced plagiarism and common problems in scientific writing [162]. The third study presented the “Group‐Effort Applied Research” (GEAR) strategy, which combined conceptual lectures with practical laboratory training, resulting in research experiences for the students [163].

Finally, the number of educational studies focusing on the inclusion of students with disabilities in biochemistry classes was low. Only one study was found, carried out by Gehret et al. [164], where evaluation was made of the effect of remote tutoring sessions on the performance and engagement of students with hearing impairments.

#### 3.2.3 Lecture‐Based Teaching Variations

This subcategory includes pedagogical practices that have been adapted from the traditional teaching format based on lectures [165, 166, 167, 168, 169, 170, 171, 172, 173, 174, 175, 176, 177, 178, 179, 180, 181, 182, 183, 184, 185]. The change from purely theoretical classes to more interactive classes is a way of altering the merely expository method and enabling greater student engagement [165, 166].

One study described how introducing scientific research history, including topics such as semi‐conservative DNA replication, in an interactive lecture format made the content accessible and relevant, in addition to arousing curiosity in the students and contributing to their development of critical thinking skills [166]. Similarly, using a detailed presentation and interactive classroom discussions, Clark [167] described how a case study on recombinant enzymes could enrich the teaching of enzyme kinetics and stereoselectivity. The results indicated that the students were able to apply theoretical concepts to a real‐life scenario, which enhanced both their understanding and interest in the subject. In a similar vein, a special topics course incorporating forensic DNA analysis and illicit drug detection was designed to stimulate student enthusiasm and enhance understanding of forensic science [178].

Several studies have explored innovative teaching strategies to enhance student engagement and performance in biochemistry. Nix [180] found that students' approaches to creative exercises varied significantly, with course modality (in‐person, online, or hybrid) influencing engagement and performance. Similarly, Nowak [182] showed that microlearning activities based on games improved comprehension and exam performance, particularly for students initially struggling with the content. Emblemsvåg [181] demonstrated that test‐enhanced learning in teams boosted engagement, motivation, and academic performance in subjects like biochemistry.

Additionally, Nguyen [183] used integrated metabolic maps to teach biochemical pathways, leading to greater satisfaction and increased use of the materials, though exam performance did not improve significantly. Kanin [177] introduced chemical biology applications in introductory organic chemistry through weekly assignments, which increased student interest and understanding without major curricular changes.

Among the studies identified, there was diverse implementation of group discussion [171, 172, 173, 185], which led to improvements in communication and problem‐solving skills. The practice known as team teaching, which consisted of a group of three teachers teaching together at the same time, was reported in the context of protein biosynthesis [168], with the results showing that the students found the classes more interesting and that they felt more comfortable in asking questions.

Rajappa [175] analyzed how book‐based tasks and group tutorials aided the learning of medical students at different stages, while Bouley [176] described a traditional biochemistry course incorporating practical activities in the classroom. In both cases, the results showed that interactive and varied methods were able to better meet the needs of the students, promoting greater engagement and improving academic performance.

In this context, two studies utilized clickers as an interactive teaching tool in biochemistry courses to foster engagement and improve student performance. Miles [169] incorporated clickers in both in‐person and distance education settings, demonstrating that students in both groups found the tool engaging and beneficial for learning, while Stines‐Chaumeil et al. [184] applied clickers exclusively in small‐group tutorials, resulting in improved exam scores and a reduced failure rate, with students from all abilities benefiting from increased retention and active reflection on exam questions. Both studies emphasize how the active engagement promoted by clickers leads to enhanced learning outcomes.

Finally, Tamilmani [179] compared the academic performance of medical students in face‐to‐face and online biochemistry classes during the COVID‐19 pandemic, finding that remote learning improved grades but did not surpass the effectiveness of in‐person teaching.

#### 3.2.4 Inquiry‐Based Learning

Inquiry‐based learning (IBL) is a very versatile methodology and has been used in biochemistry teaching for different purposes, according to the 22 studies located in this category [186, 187, 188, 189, 190, 191, 192, 193, 194, 195, 196, 197, 198, 199, 200, 201, 202, 203, 204, 205, 206, 207].

Notably, the study carried out by Murray [197] involved the design of activities based on the “Process Oriented Guided Inquiry Learning” approach, to provide students with learning based on the experience of reading and interpreting primary literature, using figures, tables, and data as models to explore topics, develop concepts, and apply them to new situations. During classroom activities, the students were guided to explore experimental methods and data in more detail, analyzing and drawing conclusions from the results presented.

Anwar [193] used this method for meaningful learning in laboratory practices. The students produced their own questions on the subject, and proposed and carried out an experiment to collect data and answer the questions. It was shown that the implementation of this methodology had a positive effect on the cognitive, psychomotor, and affective domains of the students. Other studies have also associated the IBL methodology with laboratory experiments [186, 187, 194, 198, 199, 200, 201, 204, 205, 206].

#### 3.2.5 Problem‐Based Learning

Among the studies analyzed, the problem‐based learning (PBL) method and its variations, such as the case study (CS) method, have been used in teaching the metabolism of amino acids, carbohydrates, lipids [208, 209], and vitamins [210], as well as the structure and function of nucleic acids [211] and the Krebs cycle [212]. This approach is generally well received by students [213] and can be applied in small groups [208, 211] or even in large groups [214]. The effectiveness of this methodology is highlighted by the results discussed by the authors, including the acquisition of specific knowledge and the promotion of generic problem‐solving skills [215], the improvement of creative thinking skills [210], the improvement of communication skills [213], and changes in the attitudes of students toward problem‐solving [208].

Xie et al. [216] and Shay et al. [217] demonstrated the adaptability of PBL to other methodologies, incorporating virtual simulation and the flipped classroom, respectively, to enrich the educational process. Hassan [218] investigated the perceptions of medical students concerning the difficulties faced in acquiring knowledge in biochemistry, physiology, and anatomy during the transition from traditional educational approaches to the problem‐based learning approach, offering valuable insights and perceptions for all teachers who wish to apply this method.

Ernawati et al. [219] explored how scaffolding‐based PBL, improved creative thinking skills in biochemistry students, finding significant enhancements in students' creativity and engagement through active problem‐solving and idea exchange.

In the articles discussing various CS [220, 221, 222, 223, 224, 225, 226, 227, 228], it was reported that the method contributed to academic performance and the understanding of the topic studied [220, 222, 223, 224, 225], increased student motivation [222], and improved knowledge retention [221]. Notably, Gallan et al. [223] described the application of CS in a medical course employing facilitators (students with previous experience in the method).

#### 3.2.6 Flipped Classroom

The flipped classroom is a teaching method that has become popular in STEM classes in recent years. This systematic review identified 17 studies in the context of teaching biochemistry at university level [229, 230, 231, 232, 233, 234, 235, 236, 237, 238, 239, 240, 241, 242, 243, 244, 245]. Some studies showed that students obtained similar results in tests, when compared to the lecture‐type teaching method, but highlighted a better perception of learning gains by the group that adopted the flipped classroom methodology [229, 230, 231, 232]. However, Gross [237] investigated the benefits of preparing for classes in the flipped classroom environment, in a biochemistry course, where the results showed that students who participated in this course format performed significantly better in exams.

Styers et al. [233] applied the method to investigate the possible benefits in the development of critical thinking skills, when associated with other active teaching methods during classroom time, obtaining satisfactory results. The flipped classroom methodology has also proven to be versatile when combined with other active methodologies [234, 235, 236, 240, 242, 244].

#### 3.2.7 Literature‐Based Learning

A total of 12 studies were identified that used literature‐based methods for teaching biochemistry [246, 247, 248, 249, 250, 251, 252, 253, 254, 255, 256, 257]. Teaching involving the reading and discussion of scientific papers was reported in several articles [246, 247, 250, 255, 256] and proved to be effective in broadening the knowledge of students regarding current scientific research methods in the field of biochemistry, as well as in stimulating the development of important academic skills, such as searching for, analyzing, and presenting scientific articles. Cicuto et al. [246] highlighted the importance of guiding students through the proposed activities, focusing on the most pertinent topics for the discipline, where the students who gave the best evaluation of the activity presented higher performance, indicating the importance of making them aware of the objectives of this learning method, right from the start of the activities.

The scientific literature has also been shown to be a valuable resource in proposing investigative activities. In the work by Resendes [251], students were given scientific articles to read, concerning the potential mechanisms for altering protein expression, and designed simple experiments to test their hypotheses. Similarly, Wang et al. [252] proposed in‐depth reading combined with experimental design, where the students, in groups, had to search for, read, and present scientific articles on a chosen topic, and, after class discussions, they had to propose an experimental design.

Reading non‐fiction books can also be useful for improving student engagement, as reported by Zuidema and Herndon [249]. One of the concerns of the authors was that the supplementary reading would overload them, but the students involved in the reading activity reported a positive contribution to their motivation to study the concepts they were working on.

#### 3.2.8 Project‐Based Learning

Several studies have explored project‐based learning (PBL) and project‐oriented learning (POL), which are two educational methodologies that differ mainly in their approach to the learning process. Among the 10 articles identified, four were categorized as PBL [258, 259, 260, 261], where the students were given autonomy to investigate, design, and conduct experiments independently. The remaining six articles [262, 263, 264, 265, 266, 267] utilized the POL approach, which is more instructor‐led, structured, and aligned with curriculum objectives, with the students being guided through a focused learning path centered on specific problems.

It should be noted that many pedagogical approaches can incorporate elements of both methodologies, sometimes making the distinction between PBL and POL ambiguous. For example, Hammerstad [264] reported a project‐based biochemistry laboratory module where students conducted an in‐depth study of a specific protein, from expression to characterization, fostering an integrated understanding of techniques and their application in a real research context. Similarly, Li et al. [261] demonstrated that project‐based learning in collaborative groups significantly enhanced the skills of biochemistry students, enabling them to develop competencies in experimental design, experiment execution, and results presentation, in a laboratory setting.

#### 3.2.9 Team‐Based Learning

Team‐based learning (TBL) has emerged as an effective strategy for developing dialog and critical thinking, especially in health courses [268, 269, 270, 271, 272, 273, 274, 275, 276, 277]. Some topics covered using TBL were glucose metabolism [268], the mechanism of action of different drugs and related enzymes [269], nucleic acid metabolism [270, 271], lipid metabolism [271], DNA and RNA [276], and medical genetics [277].

A significant challenge faced during the implementation of group activities lies in the individual assessment of students, since participation and engagement can vary substantially between individuals. To resolve this issue, Eguchi [268] proposed the implementation of peer assessment, arguing that it is an effective method for elucidating the role of each student in the group, thus encouraging early preparation for active participation. It was also pointed out that well‐prepared students had a better understanding of glucose metabolism and greater engagement in group discussions, showing the importance of providing simple summary documents for pre‐class preparation.

Phadtare et al. [269] described a team activity carried out with health students. The students were divided into teams and each received information about a drug/supplement, followed by questions and a group quiz. The approach had a high success rate and positive feedback from the students.

Building on this, a study on TBL applied in a biochemistry course analyzed student performance across different demographic groups, including gender, race/ethnicity, and generational status. The researchers found no significant differences in student performance based on these factors, suggesting that TBL can offer equitable learning outcomes for diverse student populations [272].

Further adapting TBL to the challenges presented by the COVID‐19 pandemic, a study explored the shift to online instruction and the effective integration of TBL in a virtual format. The researchers managed to replicate the key elements of the in‐person TBL experience in the online setting [273].

#### 3.2.10 Educational Product

The aim of building an educational product is to involve students in a practical way, allowing them to apply theoretical knowledge to create something concrete and useful. Five studies were identified that described the following products: blog [278], pamphlets [279], infographics [280], diagrammatic representation [281], and artistic representations [282].

Harrison [279] described the implementation of a project in which the students were responsible for developing information leaflets about diseases such as diabetes, lipid disorders, amino acid metabolism disorders, and metabolic genetic diseases. Throughout the semester, they produced the leaflets and presented the materials to people in a shelter. This experience allowed the students to apply biochemistry concepts in a meaningful way, while at the same time contributing to scientific divulgation.

By the construction of different products, students developed communication and writing skills [278, 279], obtained significant learning gains [278, 279, 280], especially among students with less prior knowledge [281], and acquired digital skills essential for scientific communication [280].

## 4 Conclusions

In conclusion, most of the identified didactic resources were associated with the use of digital technologies. Specifically, four out of the five subcategories included digital technologies, encompassing a total of 54 out of 78 studies (69%). This growing use of digital technologies reflects a global trend in higher education, driven by the popularization of Information and Communication Technologies (ICTs), which are playing a crucial role in modernizing higher education. The data revealed a wide range of teaching methodologies employed in biochemistry and related disciplines, distributed across 10 categories. The prevalence of experimental activities, with 50 articles (25.9%), highlighted the importance of practice and experimentation in the teaching of this discipline, enabling students to apply theories in practical contexts and develop essential laboratory skills. IBL and PBL also stood out, being reported in 21 (10.9%) and 22 (11.4%) articles, respectively, reflecting a growing trend in employing methods that encourage students to develop critical thinking and problem‐solving skills, which are essential for the formation of future professionals in the field of biochemistry.

Despite the development of various approaches for teaching Biochemistry topics, there remains the need to practically overcome the predominance of a teacher‐centered and expository teaching model. It is hoped that this review will assist in the professional development of educators seeking more effective strategies for their classes. A possible contribution of this review to the field lies in organizing the different possibilities and formats for teaching Biochemistry, providing a structured overview for educators who may wish to innovate their instructional approaches.

Lastly, recent complementary studies have reinforced the importance of adopting active methodologies and research‐based learning. A bibliometric analysis of Process‐Oriented Guided Inquiry Learning (POGIL) emphasizes its relevance and growing adoption across disciplines, while also highlighting the lack of standardized implementation protocols [283]. Similarly, a comprehensive systematic review of CUREs in STEM education underscores both the expansion of CUREs into areas like biochemistry and the need for greater incorporation of scientific reasoning practices [284]. In addition, a recent bibliometric study mapping the international scientific production in Biochemistry education [285] provides a comprehensive overview of the field's trajectory and current landscape. The analysis revealed a significant growth in publications over the last decade (over 70% of articles were published in the past 10 years) indicating an accelerated interest in educational research within this discipline. Despite this growth, the field remains largely concentrated in a few countries, with limited international collaboration and a strong dominance of the journal Biochemistry and Molecular Biology Education. The co‐citation analysis identified four major research clusters: visual literacy in Biochemistry, active learning methodologies, conceptual foundations of the curriculum, and research‐based learning. This bibliometric evidence reinforces the conclusions of our review, underscoring the necessity of expanding collaborative networks and integrating innovative, research‐driven strategies into Biochemistry education.

The scenario presented by these studies reinforces the importance of student‐centered approaches and practical, hands‐on activities, especially those that foster engagement with abstract biochemical concepts. Taken together, these studies reinforce the findings of our research, indicating that biochemistry education can significantly benefit from structured, student‐centered, and research‐integrated pedagogical approaches.

## Conflicts of Interest

The authors declare no conflicts of interest.

## Supporting information


**Table S1:** Information on the selected articles.

## Data Availability

The data that supports the findings of this study areis available in the Supporting Information of this article.
