# Supplementary material for: Biochemistry Learning in Higher Education: A Systematic Review on Methodologies and Teaching Resources
Source: Biochem Mol Biol Educ. 2025 Nov 22;54(1):29–48. doi: 10.1002/bmb.70027 (PMC12877998; doi:10.1002/bmb.70027)
Supplement: Supplementary file 1 — Table S1: Information on the selected articles. [file BMB-54-29-s001.pdf]

Table S1. Information on the selected articles

Title	Periodic	Authors	Year	Reference number
Bio-Organic Reaction Animations (BioORA): Student Performance, Student Perceptions, and Instructor Feedback	Biochemistry and Molecular Biology Education	Gunersel A. B. Fleming S.	2014	11
Computational Strategy for Visualizing Structures and Teaching Biochemistry	Biochemistry and Molecular Biology Education	Abreu P. A. et al.	2019	12
Using the PyMOL Application to Reinforce Visual Understanding of Protein Structures	Biochemistry and Molecular Biology Education	Rigsby R. E., Parker A. B.	2016	13
Virtual Protein Purification: A Simple Exercise to Introduce pH as a Parameter that Effects Ion Exchange Chromatography	Biochemistry and Molecular Biology Education	Clark D. D., Edwards D. J.	2018	14
Increasing Chemistry Students' Knowledge, Confidence, and Conceptual Understanding of pH using a Collaborative Computer pH Simulation	Chemistry Education Research and Practice	Watson S. W., Dubrovskiy A. V. Peters M. L.	2020	15
Using Molecular Visualization to Explore Protein Structure and Function and Enhance Student Facility with Computational Tools	Biochemistry and Molecular Biology Education	Terrell, C. R. Listenberger L. L.	2017	16
Assessment of Students' External Representations of mmCIF Entries and their Biochemical Knowledge	Biochemistry and Molecular Biology Education	Ealy J. B.	2018	17
A Survey of Educational Uses of Molecular Visualization Freeware	Biochemistry and Molecular Biology Education	Craig P. A. Michel L. V. Bateman R. C.	2013	18
Molecular Dynamics Characterization of the Conformational Landscape of Small Peptides: A Series of Hands-on Collaborative Practical Sessions for Undergraduate Students	Biochemistry and Molecular Biology Education	Rodrigues J. P.G.L.M. Melquiond A. S.J. Bonvin A.M.J.J.	2016	19
Implementing a Web-Based Introductory Bioinformatics Course for Non-Bioinformaticians That Incorporates Practical Exercises	Biochemistry and Molecular Biology Education	Vincent A. T. et al.	2018	20

Exploration of an Interactive “Virtual and Actual Combined” Teaching Mode in Medical Developmental Biology	Biochemistry and Molecular Biology Education	Xu X. et al.	2018	21
Qualitative Assessment of a 3D Simulation Program: Faculty, Students, and Bio-Organic Reaction Animations	Journal of Chemical Education	Günersel A. B, Fleming S. A.	2013	22
Integrative Teaching of Metabolic Modeling and Flux Analysis with Interactive Python Modules	Biochemistry and Molecular Biology Education	Kaste J. A. M., Green A., Shachar-Hill Y.	2023	23
PeerWise Provides Significant Academic Benefits to Biological Science Students Across Diverse Learning Tasks, But with Minimal Instructor Intervention	Biochemistry and Molecular Biology Education	McQueen H. A. et al.	2014	24
The Usability of WeChat as a Mobile and Interactive Medium in Student-Centered Medical Teaching	Biochemistry and Molecular Biology Education	Wang J. et al.	2017	25
Use of Rain Classroom as a Teaching Tool in a Biochemistry Course	Journal of Curriculum and Teaching	Shu B., Fan F., Zhu1 X.	2019	26
Application effect evaluation of hybrid biochemistry teaching model based on WeChat platform under the trend of COVID-19	Medicine	Ding J. et al.	2023	27
Student Use and Pedagogical Impact of a Mobile Learning Application	Biochemistry and Molecular Biology Education	Teri S., et al.	2014	28
Benefits and Challenges of a Virtual Laboratory in Chemical and Biochemical Engineering: Students’ Experiences in Fermentation	Journal of Chemical Education	Canõ De Las Heras et al.	2021	29
Implementation and Evaluation of a Three-Dimensional Virtual Reality Biology Lab versus Conventional Didactic Practices in Lab Experimenting with the Photonic Microscope	Biochemistry and Molecular Biology Education	Paxinou E. et al.	2020	30
Teaching Structure: Student Use of Software Tools for Understanding Macromolecular Structure in an Undergraduate Biochemistry Course	Biochemistry and Molecular Biology Education	Jaswal S. S. et al.	2013	31
An Integrated Microbiome Project for Charactering Microbial Diversity in Classroom Based on Virtual Simulation Experiments	Biochemistry and Molecular Biology Education	Sun H., Wang P., Li Y.	2023	32

Teaching the Fluctuation Test "In Silico" by Using Mutate: A Program to Distinguish between the Adaptive and Spontaneous Mutation Hypotheses	Biochemistry and Molecular Biology Education	Carvajal-Rodriguez A.	2012	33
Assessing the Engagement, Learning, and Overall Experience of Students Operating an Atomic Absorption Spectrophotometer with Remote Access Technology	Biochemistry and Molecular Biology Education	Erasmus D. J. Brewer S. E. Cinel B.	2015	34
Active learning tools improve the learning outcomes, scientific attitude, and critical thinking in higher education: Experiences in an online course during the COVID-19 pandemic	Biochemistry and Molecular Biology Education	.Rossi I. V. et al.	2021	35
Aiming for the Bullseye: Targeted activities decrease misconceptions related to enzyme function for undergraduate biochemistry students	Biochemistry and Molecular Biology Education	Terrell, C. R.; et al.	2021	36
Physical Models Can Provide Superior Learning Opportunities Beyond the Benefits of Active Engagements	Biochemistry and Molecular Biology Education	Newman, D. L.; et al.	2018	37
Creating 3D Physical Models to Probe Student Understanding of Macromolecular Structures	Biochemistry and Molecular Biology Education	Cooper A. K.; Oliver-Hoyo M. T.	2017	38
Building Mental Models by Dissecting Physical Models	Biochemistry and Molecular Biology Education	Srivastava, A.	2016	39
Student Understanding of DNA Structure–Function Relationships Improves from Using 3D Learning Modules with Dynamic 3D Printed Models	Biochemistry and Molecular Biology Education	Howell M. E.; et al.	2019	40
Active learning in the lecture theatre using 3D printed objects	F1000Research	Smith D. P	2016	41
Two Active Learning Models of Protein Dynamics for Use in Undergraduate Biochemistry Courses	Journal of Chemical Education	Theisen K. E.	2022	42
Interactive learning modules with 3D printed models improve student understanding of protein structure–function relationships	Biochemistry and Molecular Biology Education	Howell M. E.; et al.	2020	43
Physical Models Have Gender-Specific Effects on Student Understanding of Protein Structure–Function Relationships	Biochemistry and Molecular Biology Education	Forbes-Lorman, R. M. et al.	2016	44

Biochemist-Tree: Using Modular Origami to Understand the Integration of Intermediary Metabolism	Biochemistry and Molecular Biology Education	Sharp D.	2013	45
Using simple manipulatives to improve student comprehension of a complex biological process: Protein synthesis	Biochemistry and Molecular Biology Education	Guzman K.; Bartlett J.	2012	46
Pop-It Beads to Introduce Catalysis of Reaction Rate and Substrate Depletion Effects	Biochemistry and Molecular Biology Education	Gehret A. U.	2017	47
Case study: Perspectives on the use of LEGO® bricks in the biochemistry classroom"	Essays in Biochemistry	Austin S., Millar C., Christmas S.	2022	48
The Polygonal Model: A Simple Representation of Biomolecules as a Tool for Teaching Metabolism	Biochemistry and Molecular Biology Education	Bonafe, C. F. S. Bispo, J. A. C. Jesus, M. B.	2018	49
Teaching Structural Diversity of Hexoses to Graduate and Postgraduate Students: Methods to Correlate Stereochemistry	Biochemistry and Molecular Biology Education	Arya A., Kumar A,	2020	50
Game-based activities targeting visual literacy skills to increase understanding of biomolecule structure and function concepts in undergraduate biochemistry	Biochemistry and Molecular Biology Education	Terrell, C. R.; et al.	2021	51
Amino-structure: A Card Game for Amino Acids Learning in Biochemistry Classes	Journal of Chemical Education	Gomez Buitrago, P.; Tobar-Munoz, H.; Arteaga, D.	2024	52
CARBGame (CARd & Board GAmes in Medical Education): A Gamification Innovation to Foster Active Learning in Biochemistry for Medical Students	Advances in Physiology Education	Surapaneni, K. M.	2024	53
Livogena: The Ikteros Curse-A Jaundice Narrative Card and Board Game for Medical Students	Journal of Teaching and Learning Resources	Surapaneni, K. M.	2024	54
Interactive Hangman Teaches Amino Acid Structures and Abbreviations	Biochemistry and Molecular Biology Education	Pennington B. O.; Sears D.; Clegg D.O.	2014	55
Utility of Self-Made Crossword Puzzles as an Active Learning Method to Study Biochemistry in Undergraduate Education	Journal of College Science Teaching	Coticone S. R.	2013	56

DNA Re-EvolutioN: A Game for Learning Molecular Genetics and Evolution	Biochemistry and Molecular Biology Education	Miralles, L.; et al.	2013	57
Interactive Metabolism, a simple and robust active learning tool that improves the biochemistry knowledge of undergraduate students	Advances in physiology education	Campos, W. F.; França, V. C.	2020	58
Teaching meiosis with the DNA triangle framework: A classroom activity that changes how students think about chromosomes	Biochemistry and Molecular Biology Education	Wright L. K.; Cortez P.; Franzen M. A.	2022	59
"N.A.M.E." FUN! Emojis may illustrate structure-function relationships of neurotransmitters to health professions students	Advances in Physiology Education	Mahaffey A. L.	2021	60
Responsive eLearning Exercises to Enhance Student Interaction with Metabolic Pathways	Biochemistry and Molecular Biology Education	Roesler W. J. Dreaver-Charles K.	2018	61
A mobile technology-based cooperative learning platform for undergraduate biology courses in common college classrooms	Biochemistry and Molecular Biology Education	Lewis M., Zhou C.	2021	62
Online Preparation Resources Help First Year Students to Benefit from Practical Classes	Biochemistry and Molecular Biology Education	Bickerdikeb S. R. Whittle S. R.	2014	63
Using pre-lecture activities to enhance learner engagement in a large group setting	Active Learning in Higher Education	Kinsella G. K. Mahon C. Lillis S.	2017	64
An Online Guided e-Journal Exercise in Pre-Clerkship Years: Oxidative Phosphorylation in Brown Adipose Tissues	Biochemistry and Molecular Biology Education	Abali E. E. et al.	2014	65
Overcoming the Challenges of Remote Instruction: Using Mobile Technology to Promote Active Learning	Journal of Chemical Education	Baldock, B. L. et al.	2021	66
Creating Custom Foldit Puzzles for Teaching Biochemistry	Biochemistry and Molecular Biology Education	Dsilva L. et al.	2019	67
"Discovering a Glycoprotein: The Case of the H,K-ATPase". An Online Game for Improvement of Reading Skills in a Course of Biological Chemistry	Journal of Chemical Education	Valsecchi, W. M.; Dominici, F. P.; Gomez, K. A.	2023	68
Lessons Learned from Active Engagement in a Large-Enrollment Introductory Biochemistry Course during a Remote Quarter.	Journal of Chemical Education	Dingwall, S.	2020	69

Online Research Immersion Program to Increase Introductory Science Student Confidence and Science Identity.	Biochemistry and Molecular Biology Education	Zheng, E. J.; Oakes, E.; Roden, J.	2024	70
An Interdisciplinary Course on Computer-Aided Drug Discovery to Broaden Student Participation in Original Scientific Research.	Biochemistry and Molecular Biology Education	Stratton, C. et al.	2024	71
Learning lab skills online: Lessons from implementing video-based instruction for a remote biotechnology lab	Biochemistry and Molecular Biology Education	Chen S. H.	2022	72
Audio–Visual Aid in Teaching "Fatty Liver"	Biochemistry and Molecular Biology Education	Dash et al	2016	73
Use of Short Videos and Case Studies to Enhance Student Confidence in Biochemistry Knowledge and Application in a Large Lecture Biochemistry Course in First Year Veterinary Curriculum.	Biochemistry and Molecular Biology Education	Cardamone, C. et al.	2023	74
Amino Acid Jazz: Amplifying Biochemistry Concepts with Content- Rich Music"	Journal of Chemical Education	Crowther G. J. Davis K.	2013	75
Meal–Insulin Cycle: A visual summary of the biochemical events between meals	Biochemistry and Molecular Biology Education	Kalogiannis S.	2016	76
Metabolism in Motion: Engaging Biochemistry Students with Animation	Journal of Chemical Education	Long, S. et al.	2021	77
What do biochemistry students pay attention to in external representations of protein translation? The case of the Shine–Dalgarno sequence	Chemistry Education Research and Practice	Bussey T. J., Orgill M.	2015	78
Using Metro Lines for Integration of Nucleotide Metabolic Pathways	Biochemistry and Molecular Biology Education	Lee R. K. Y. Ng B. Y. N. Chen D. M. H.	2019	79
Adoption of the ADDIE Approach in an Agile Way for the Development of Biochemistry Courseware for Learning Metabolism	Journal of Chemical Education	Lee R. K. Y. Ng B. Y. N. Chen D. M. H.	2024	80
Variation in External Representations as Part of the Classroom Lecture: An Investigation of Virtual Cell Animations in Introductory Photosynthesis Instruction	Biochemistry and Molecular Biology Education	Goff E. E. et al.	2017	81

Design, Implementation, and Assessment of an Interactive Simulation to Teach Undergraduate Immunology Students Hemolytic Disease of the Newborn	Advances in Physiology Education	Costabile, M.	2021	82
Exploring Chemistry with Wireless, PC-Less Portable Virtual Reality Laboratories	Journal of Chemical Education	Qin T. Cook M., Courtney M.	2021	83
Teaching Biochemistry and Molecular Biology With Virtual Reality — Lesson Creation and Student Response	Journal of Teaching and Learning	Coan, H. A.; Goehle, G.; Youker, R. T.	2020	84
Investigating the effect of teaching as a generative learning strategy when learning through desktop and immersive VR: A media and methods experiment	British Journal of Educational Technology	Klingenberg, S. et al.	2020	85
Teaching Enzyme Catalysis Using Interactive Molecular Dynamics in Virtual Reality	Journal of Chemical Education	Bennie, S. J. et al.	2019	86
An idea to explore: Use of augmented reality for teaching three-dimensional biomolecular structures	Biochemistry and Molecular Biology Education	Peterson, C. N.; et al.	2020	87
Fast, Simple, Student Generated Augmented Reality Approach for Protein Visualization in the Classroom and Home Study	Journal of Chemical Education	Argüello J. M. Dempski R. E.	2020	88
A Ten-Week Biochemistry Lab Project Studying Wild-Type and Mutant Bacterial Alkaline Phosphatase	Biochemistry and Molecular Biology Education	Witherow D. S.	2016	90
Simulated Sandwich Enzyme-Linked Immunosorbent Assay for a Cost-Effective Investigation of Natural and Engineered Cellular Signaling Pathways	Biochemistry and Molecular Biology Education	Jaschke P. R.	2020	91
Purification and Electrophoretic Characterization of Lactate Dehydrogenase from Mammalian Blood: A Different Twist on a Classic Experiment	Journal of Chemical Education	Brunauer L. S.	2016	92
Using NMR Spectroscopy To Measure Protein Binding Capacity on Gold Nanoparticles	Journal of Chemical Education	Perera Y. R. et al.	2020	93
Using an FPLC to Promote Active Learning of the Principles of Protein Structure and Purification	Biochemistry and Molecular Biology Education	Robinson R. L.	2017	94

Doing that Thing That Scientists Do: A Discovery-Driven Module on Protein Purification and Characterization for the Undergraduate Biochemistry Laboratory Classroom	Biochemistry and Molecular Biology Education	Garrett T. A et al.	2015	95
Linking Biochemistry Concepts to Food Safety<br>Using Yogurt as a Model	Journal of Food Science Education	Zimmerman T.	2019	96
Aqueous Biphasic Systems in the Separation of Food Colorants	Biochemistry and Molecular Biology Education	Santos J. H. P. M. et al.	2018	97
Circular dichroism spectroscopy: Enhancing a traditional undergraduate biochemistry laboratory experience	Biochemistry and Molecular Biology Education	Lewis, R. L. et al.	2017	98
A Laboratory Class: Constructing DNA Molecular Circuits for Cancer Diagnosis.	Journal of Chemical Education	Bardales, A. C.; Vo, Q.; Kolpashchikov, D. M.	2024	99
Assessment of α-Amylase Activity in a Microanalysis System: Experimental Optimization and Evaluation of Type of Inhibition.	Journal of Chemical Education	Freitas, M. et al.	2023	100
Food Control and a Citizen Science Approach<br>for Improving Teaching of Genetics in Universities	Biochemistry and Molecular Biology Education	Borrell Y. J. et al.	2016	101
Practices and Exploration on Competition of Molecular Biological Detection Technology Among Students in Food Quality and Safety Major	Biochemistry and Molecular Biology Education	Chang Y. et al.	2017	102
Teaching Practice on the Anaerobic Degradation of Biodegradable Plastic Products.	Journal of Chemical Education	Chen, C.; Jin, Y.; Liu, G.	2024	103
Two-Dimensional Nuclear Magnetic Resonance<br>Structure Determination Module for Introductory<br>Biochemistry: Synthesis and Structural Characterization of Lyso-Glycerophospholipids	Biochemistry and Molecular Biology Education	Garret T. A.<br>Rose R. L.<br>Bell S. M.	2013	104
Characterizing biological macromolecules with attenuated total reflectance–Fourier transform infrared spectroscopy provides hands-on spectroscopy experiences for undergraduates	Biochemistry and Molecular Biology Education	Helburn R.<br>Nolan K.	2022	105

CRISPR in butterflies: An undergraduate lab experience to inactivate wing patterning genes during development	Biochemistry and Molecular Biology Education	Thulluru A. et al.	2022	106
An Undergraduate Laboratory Class Using CRISPR/Cas9 Technology to Mutate Drosophila Genes	Biochemistry and Molecular Biology Education	Adame V. et al.	2016	107
An Undergraduate Laboratory Module that Uses the CRISPR/Cas9 System to Generate Frameshift Mutations in Yeast	Biochemistry and Molecular Biology Education	De Waal et al.	2019	108
An undergraduate laboratory to detect viruses in human DNA samples using qPCR	Biochemistry and Molecular Biology Education	Militello K. T. Nedelkovska H.	2022	109
Random Amplified Polymorphic DNA PCR in the Teaching of Molecular Epidemiology	Biochemistry and Molecular Biology Education	Reinoso E. B. Bettera S. G.	2016	110
Hydrodistillation and antimicrobial properties of lemongrass oil (Cymbopogon citratus, Stapf): An undergraduate laboratory exercise bridging chemistry and microbiology	Journal of Food Science Education	Dangkulwanich M. Charaslertrangsi T.	2020	111
A Model System for the Study of Gene Expression in the Undergraduate Laboratory	Biochemistry and Molecular Biology Education	Hargadon K. M.	2016	112
Separation of mononuclear cells and identification of B lymphocytes: A comprehensive experiment for medical students	Biochemistry and Molecular Biology Education	Li J. et al.	2020	113
Protein Colorimetry Experiments That Incorporate Intentional Discrepancies and Historical Narratives	Journal of Chemical Education	Astrof N. S. Horowitz G.	2018	114
An Adaptable Investigative Graduate Laboratory Course for Teaching Protein Purification.	Biochemistry and Molecular Biology Education	Carroll, C. W.; Keller, L. C.	2014	115
Exploration of simulated urine sample biochemistry for the diagnosis of diseases: A laboratory practical exercise	Biochemistry and Molecular Biology Education	Denis C. Lasfargues C. Buffin-Meyer B.	2021	116
Teaching Enzymes to Pre-service Science Teachers through POE (Predict, Observe, Explain) Method: The Case of Catalase	Asia-Pacific Forum on Science Learning and Teaching	Gungor S. N. Ozkan M.	2016	117

Relating Chemistry to Healthcare and MORE: Implementation of MORE in a Survey Organic and Biochemistry Course for Prehealth Students	Journal of Chemical Education	Schroeder L. et al.	2017	118
The Use of Orientation/Decision/Do/ Discuss/Reflect (Od3r) Method to Increase Critical Thinking Skill and Practical Skill in Biochemistry Learnings	Biochemistry and Molecular Biology Education	Anwar Y. A. S. Senam Laksono E. W.	2018	119
Meaningful Biochemistry Learning Using the Orientation-Decision-Do-Discuss-Reflect (OD3R) Method	International Journal of Instruction	Anwar Y. A. S. Senam Laksono E. W.	2018	120
Effective Laboratory Work in Biochemistry Subject: Students' and Lecturers' Perspective in Indonesia	International Journal of Higher Education	Anwar Y. A. S. Senam Laksono E. W.	2017	121
Expression, Purification, and Characterization of a Carbohydrate-Active Enzyme: A Research-Inspired Methods Optimization Experiment for the Biochemistry Laboratory	Biochemistry and Molecular Biology Education	Willbur et al.	2016	122
Understanding new molecular and cell biology findings based on progressives cientific practices and interconnected Activities in undergraduate student	Biochemistry and Molecular Biology Education	Giojalas et al.	2020	123
Lab experience with sea food control at the undergraduate level: Cephalopods as a case study	Biochemistry and Molecular Biology Education	Fernández et al.	2020	124
Student Perceptions of Their Gains in Course-Based Undergraduate Research Abilities Identified as the Anticipated Learning Outcomes for a Biochemistry CURE	Journal of Chemical Education	Irby S. M. Pelaez N. J. Anderson T. R.	2020	125
The Beer and Biofuels Laboratory: A Report on Implementing and Supporting a Large, Interdisciplinary, Yeast-Focused Course-Based Undergraduate Research Experience	Biochemistry and Molecular Biology Education	Pedwell R. K. et al.	2018	126
Stressing Escherichia coli to Educate Students About Research: A CURE to Investigate Multiple Levels of Gene Regulation	Biochemistry and Molecular Biology Education	McDonough J. et al.	2017	127
Making it Stick: A CURE Designed to Introduce Students to the Scientific Process and the Host Response to Foreign Materials	Biochemistry and Molecular Biology Education	Slee J. B. McLaughlin J. S.	2019	128

Cancer and Chemicals: A Research-Inspired Laboratory Exercise Based on the Ames Test for Mutagenicity.	Biochemistry and Molecular Biology Education	Large, D. N.; Van Doorn, N. A.; Timmons, S. C.	2023	129
Identifying New Small Proteins through a Molecular Biology Course-Based Undergraduate Research Experience Laboratory Class	Biochemistry and Molecular Biology Education	Miranda, R. J. et al	2023	130
Continuous In Vitro Evolution of a Ribozyme Ligase: A Model Experiment for the Evolution of a Biomolecule	Biochemistry and Molecular Biology Education	Ledbetter M. P. et al.	2013	131
Immersing Undergraduate Students in the Research Experience	Biochemistry and Molecular Biology Education	Wang J. T. H. et al.	2012	132
Identification of an Unknown Glycoprotein from Whole Cell Lysate Using conA and Mass Spectrometry	Biochemistry and Molecular Biology Education	Yan J. et al.	2018	133
From genetics to biotechnology: Synthetic biology as a flexible course-embedded research experience	Biochemistry and Molecular Biology Education	Johnson K. C. et al.	2022	134
An Undergraduate Laboratory Experience Using CRISPR-cas9 Technology to Deactivate Green Fluorescent Protein Expression in Escherichia coli	Biochemistry and Molecular Biology Education	Pieczynski J. N. et al.	2019	135
A Modular Laboratory Course Using Planarians to Study Genes Involved in Tissue Regeneration	Biochemistry and Molecular Biology Education	Ochoa S. D. et al.	2019	136
A Course-Based Undergraduate Research Experience in Biochemistry that is Suitable for Students with Various Levels of Preparedness	Biochemistry and Molecular Biology Education	Shelby S. J.	2019	137
Continuous improvement engineering: Preparing students for changing world	Biochemistry and Molecular Biology Education	Mishra et al.	2022	138
Is the Undergraduate Research Experience (URE) Always Best?	Biochemistry and Molecular Biology Education	Rowland S. L. et al.	2012	139
Peer Teaching Experience of the First Year Medical Students from Turkey	Journal of the College of Physicians and Surgeons Pakistan	Cansever Z. et al.	2015	140

Embedding Retrieval Practice in Undergraduate Biochemistry Teaching Using Peerwise	Biochemistry and Molecular Biology Education	Higgins, T. et al.	2024	141
Figure Analysis: A Teaching Technique to Promote Visual Literacy and Active Learning	Biochemistry and Molecular Biology Education	Wiles A. M.	2016	142
Improving Large Class Performance and Engagement Through Student-Generated Question Banks	Biochemistry and Molecular Biology Education	Hancock D. et al.	2018	143
Improving student understanding of lipids concepts in a biochemistry course using test-enhanced learning	Chemistry Education Research and Practice	Horn S. Hernick M.	2015	144
Individual Identification and Correction of Mistakes in Statements of Biochemical Significance: An Effective Learning Process for Graduate Medical Students	Biochemistry and Molecular Biology Education	Bobby Z. et al.	2019	145
Understanding Student Characteristics in the Development of Active Learning Strategies	Medical Science Educator	Mehta S. et al.	2022	146
Diverse Assessment and Active Student Engagement Sustain Deep Learning: A Comparative Study of Outcomes in Two Parallel Introductory Biochemistry Courses	Biochemistry and Molecular Biology Education	Bevan S. J. Chan C. W. L. Tenner J. A.	2014	147
Blended Learning in Biochemistry Education: Analysis of Medical Students' Perceptions.	Biochemistry and Molecular Biology Education	De Fátima Wardenski et al.	2012	148
Students' use of chemistry core ideas to explain the structure and stability of DNA	Biochemistry and Molecular Biology Education	Roche Allred et al.	2020	149
Comparison of Two Different Techniques of Cooperative Learning Approach: Undergraduates' Conceptual Understanding in the Context of Hormone Biochemistry	Biochemistry and Molecular Biology Education	Mutlu A.	2018	150
Community-Based Inquiry in Allied Health Biochemistry Promotes Equity by Improving Critical Thinking for Women and Showing Promise for Increasing Content Gains for Ethnic Minority Students	Journal of Chemical Education	Goeden et al.	2015	151
Application of Mini-Clinical Evaluation Exercise for Assessing the Integrated-Based Learning During Physical Diagnostic Course	Biochemistry and Molecular Biology Education	Da et al.	2018	152

Application of Ausubel cognitive assimilation theory in teaching/learning medical biochemistry and Molecular biology	Biochemistry and Molecular Biology Education	Tian et al.	2020	153
Genomics Course Design and Combined Teaching Strategy to Enhance Learning Initiatives in Classroom	Biochemistry and Molecular Biology Education	Ouyang et al.	2019	154
Participation of Undergraduate Students in a Controlled Feeding Study with Metabolomics Analysis to Enhance Learning of Metabolism	Journal of Chemical Education	La Frano M. R. Amin S. Fanter R. K.	2020	155
Effectiveness of an “Online + In-Person” Hybrid Model for an Undergraduate Molecular Biology Lab during COVID-19	Biochemistry and Molecular Biology Education	Sun, Z. et al.	2023	156
Improving Self-Directed Learning Ability of Medical Students Using the Blended Teaching Method: A Quasi-Experimental Study	BMC Med Educ	Lu, S. Y. et al.	2023	157
A. Structure and Function of Biomacromolecules: A Chemistry/Biochemistry Transdisciplinary Workshop	Biochemistry and Molecular Biology Education	Sancassani, S. et al.	2024	158
The Use of an Imagery Mnemonic to Teach the Krebs Cycle	Biochemistry and Molecular Biology Education	Morisaki R, Bon C. Levitt J. O.	2016	159
Using Analogy Role-Play Activity in an Undergraduate Biology Classroom to Show Central Dogma Revision	Biochemistry and Molecular Biology Education	Takemura M. Kurabayashi M.	2014	160
“On the Job” Learning: A Bioinformatics Course Incorporating Undergraduates in Actual Research Projects and Manuscript Submissions	Biochemistry and Molecular Biology Education	Smith J. T et al.	2015	161
Writing in Your Own Voice: An Intervention that Reduces Plagiarism and Common Writing Problems in Students’ Scientific Writing	Biochemistry and Molecular Biology Education	Yang A. Stockwell S. McDonnell L.	2019	162
Group-effort Applied Research: Expanding Opportunities for Undergraduate Research Through Original, Class-Based Research Projects	Biochemistry and Molecular Biology Education	Moore S. D. Teter K.	2014	163
Active Collaborative Learning Through Remote Tutoring: A Case Study With Students Who Are Deaf or Hard of Hearing	Biochemistry and Molecular Biology Education	Gehret A. U. Elliot L. B. MacDonald J. H. C.	2017	164

Medical biochemistry: Is it time to change the teaching style?	The National Medical Journal of India	Palocaren, J., Pillai, L. S., TM, C.	2016	165
Incorporation of classical scientific research stories into traditional lecture classes to promote the active learning of students	Biochemistry and Molecular Biology Education	Yang X. et al.	2021	166
Characterization of the Recombinant (R)- and (S)-Hydroxypropyl-Coenzyme M Dehydrogenases: A Case Study to Augment the Teaching of Enzyme Kinetics and Stereoselectivity	Biochemistry and Molecular Biology Education	Clark D. D.	2019	167
Effectiveness of team teaching in biochemistry lectures for undergraduate students	Biochemistry and Molecular Biology Education	Govindarajan S. et al.	2021	168
Acceptance of Clickers in a Large Multimodal Biochemistry Class as Determined by Student Evaluations of Teaching: Are They Just an Annoying Distraction for Distance Students?	Biochemistry and Molecular Biology Education	Miles N. G. Costa T. P. S	2016	169
FastFeedback Questions: A New Teaching Method	Biochemistry and Molecular Biology Education	Elnashar M. M.	2018	170
Modified Conventional Teaching: An Assessment of Clinical Biochemistry Learning Process Among Medical Undergraduate Students Using the traditional Teaching in Combination with Group Discussion	Cureus	Vadakedath S. Kandi V.	2019	171
Engaging Pharmacy Students in Interactive Life-Based Situations as the Basis for Teaching a Biochemistry Course	Cureus	Al-Gayyar M. M.	2020	172
Implementing an Active Learning Environment To Influence Students' Motivation in Biochemistry	Journal of Chemical Education	Cicuto C. A. T. Torres B. B.	2016	173
Student Performance in a Seminar Based Examination of Basic Biochemistry Course	Chemistry: Bulgarian Journal of Science Education	Mohammed M. E. A.	2015	174
Using Optimal Combination of Teaching–Learning Methods (Open Book Assignment and Group Tutorials) as Revision Exercises to Improve Learning Outcome in Low Achievers in Biochemistry	Biochemistry and Molecular Biology Education	Rajappa M. et al.	2016	175

Redesigning a One-Semester Biochemistry Class Using In-Class Activities to Achieve High Student Engagement	Journal of Chemical Education	Bouley R.	2022	176
Introducing Chemical Biology Applications to Introductory Organic Chemistry Students Using Series of Weekly Assignment	Biochemistry and Molecular Biology Education	Kanin, M. R.; Pontrello, J. K	2016	177
DNA, Drugs, and Detectives: An Interdisciplinary Special Topics Course for Undergraduate Students in Forensic Science	Journal of College Science Teaching	Coticone, S. R.; Van Houten, L. B.	2015	178
Comparison of academic performance of medical undergraduate students between routine class room teaching and online assisted teaching in biochemistry during COVID pandemic	Biochemistry and Molecular Biology Education	Tamilmani, K. et al.	2023	179
Exploration of Student Approaches to Creative Exercises in Undergraduate Biochemistry	Journal of Chemical Education	Nix, C. A.; Hughes, H.; Saitta, E. K. H.	2023	180
Nursing students' experiences with test-enhanced learning in teams: A cross-sectional study	Nurse Educ Today	Emblemsvåg, M. S.	2024	181
Microlearning activities improve student comprehension of difficult concepts and performance in a biochemistry course	Curr Pharm Teach Learn	Nowak, G.; Speed, O.; Vuk, J.	2023	182
Use of Integrated Metabolic Maps as a Framework for Teaching Biochemical Pathways in the Pre-clinical Medical Curriculum	Med Sci Educ	Nguyen, K.; Silveira, J. R.; Lounsbury, K. M.	2024	183
Incorporating clickers into an enzymology course improves student performance.	FEBS Open Bio	Stines-Chaumeil, C.; Paumard, P.; Hooks, M. A.	2019	184
The Science of Human Health: A Context-Based Chemistry Course for Non-Science Majors Incorporating Systems Thinking	Journal of Chemical Education	Armstrong, D.; Poe, J. C.	2020	185
Inquiry-Based Experiments for Large-Scale Introduction to PCR and Restriction Enzyme Digests	Biochemistry and Molecular Biology Education	Johanson K. E. Watt T. J.	2015	186
The Use of Biotin to Demonstrate Immunohistochemistry, Western Blotting, and Dot Blots in University Practical Classes	Biochemistry and Molecular Biology Education	Millar T. J. Knighton R. Chuck J.	2012	187

Effectiveness and perceptions of MBBS students about process-oriented guided inquiry learning in biochemistry	The National Medical Journal of India	Sonoli S. S. Sankanagoudar S.	2020	188
Guided Inquiry Activities for Learning about the Macro- and Micronutrients in Introductory Nutrition Courses	Biochemistry and Molecular Biology Education	Smith A. L Purcell R. J. Vaughan J. M	2015	189
Learning Transferable Skills in Large Lecture Halls: Implementing a POGIL Approach in Biochemistry	Biochemistry and Molecular Biology Education	Bailey C. P. Minderhout V. Loertscher J.	2012	190
Not all Has Been Said about Glucose Oxidase/Peroxidase: New Pedagogical Uses for a Classical and Robust Undergraduate Laboratory Experiment	Biochemistry and Molecular Biology Education	García-Ponce A. L. et al.	2019	191
The Genetic Code Kit: An Open-Source Cell-Free Platform for Biochemical and Biotechnology Education	Frontiers in Bioengineering and Biotechnology	Williams L. C. et al.	2020	192
The Multilevel Inquiry Approach to Achieving Meaningful Learning in Biochemistry Course	Biochemistry and Molecular Biology Education	Anwar Y. A. S.	2020	193
Developing and Supporting Students' Autonomy To Plan, Perform, and Interpret Inquiry-Based Biochemistry Experiments	Journal of Chemical Education	Silva T. Galambeck E.	2016	194
Exploring the Inquiry-Based Learning Structure to Promote Scientific Culture in the Classrooms of Higher Education Sciences	Biochemistry and Molecular Biology Education	Mello P. S. et al.	2019	195
Effects of Guided Inquiry versus Lecture Instruction on Final Grade Distribution in a One-Semester Organic and Biochemistry Course	Journal of Chemical Education	Conway C. J.	2014	196
Teaching Students to Read the Primary Literature Using POGIL Activities	Biochemistry and Molecular Biology Education	Murray T. A.	2014	197
A guided-inquiry investigation of genetic variants using Oxford nanopore sequencing for an undergraduate molecular biology laboratory course	Biochemistry and Molecular Biology Education	Rodriguez P. D. et al.	2021	198
Inquiry-Based Laboratories for Students to Investigate the Concepts of Acid-Base Titration, pK[Subscript a], Equivalence Points, and Molar Absorption Coefficients.	Journal of Chemical Education	Pinthong, C. et al.	2022	199

An inquiry-based approach in large undergraduate labs: Learning, by doing it the “wrong” way	Biochemistry and Molecular Biology Education	Bachhawat A. K. et al.	2020	200
Integration of a Faculty’s Ongoing Research into an Undergraduate Laboratory Teaching Class in Developmental Biology	Biochemistry and Molecular Biology Education	Nam S.	2018	201
Writing Throughout the Biochemistry Curriculum: Synergistic Inquiry-Based Writing Projects for Biochemistry Students	Biochemistry and Molecular Biology Education	Mertz P. Streu C.	2015	202
The impact of semester-long authentic research on student experiences	Journal of Biological Education	Fornsaglio J. L et al.	2019	203
An authentic inquiry-based laboratory module for introducing concepts about volatile-mediated communication resulted in stronger students' self-efficacy	Biochemistry and Molecular Biology Education	Chiang C. et al.	2021	204
An Inquiry-Based Biochemistry Laboratory Structure Emphasizing Competency in the Scientific Process: A Guided Approach with an Electronic Notebook Format	Biochemistry and Molecular Biology Education	Hall M. L. Vardar-Ulu D.	2014	205
An Inquiry-based Practical for a Large, Foundation-Level Undergraduate Laboratory that Enhances Student Understanding of Basic Cellular Concepts and Scientific Experimental Design	Biochemistry and Molecular Biology Education	Bugarcic A. et al.	2012	206
Evaluating the Effectiveness of a Practical Inquiry-Based Learning Bioinformatics Module on Undergraduate Student Engagement and Applied Skills	Biochemistry and Molecular Biology Education	Brown J. A. L	2016	207
The Views of Undergraduates about Problem-based Learning Applications in a Biochemistry Course	Journal of Biological Education	Tarhan L. Ayyıldız Y.	2014	208
A problem-/case-based learning approach as an useful tool for studying glycogen metabolism and its regulation	Biochemistry and Molecular Biology Education	García-Ponce A. L. et al	2020	209
Analysis of problem based learning in the scaffolding design: Students’ creative-thinking skills	Cypriot Journal of Educational Sciences	Ernawati M. D. W. et al.	2022	210
Learning Nucleic Acids Solving by Bioinformatics Problems	Biochemistry and Molecular Biology Education	Nunes R et al.	2015	211

The Evolution of the Krebs Cycle: A Promising Subject for Meaningful Learning of Biochemistry	Biochemistry and Molecular Biology Education	Costa C. Galembeck E.	2016	212
A Comparison of Debate and Role Play in Enhancing Critical Thinking and Communication Skills of Medical Students During Problem Based Learning"	Biochemistry and Molecular Biology Education	Latif R. et al.	2018	213
Improved Performance of Students Instructed in a Hybrid PBL Format	Biochemistry and Molecular Biology Education	Lian J. He F.	2013	214
Improvement in Generic Problem-Solving Abilities of Students by Use of Tutor-less Problem-Based Learning in a Large Classroom Setting	CBE - Life Sciences Education	Klegeris A., Bahniwal M Hurren H.	2013	215
Application of problem-based learning combined with a virtual simulation training platform in clinical biochemistry teaching during the COVID-19 pandemic	Frontiers in medicine	Xie H. et al.	2022	216
Tackling Real-World Environmental Paper Pollution: A Problem-Based Microbiology Lesson About Carbon Assimilation	Frontiers in Microbiology	Shay J. E. Solis R. García, M. E.	2020	217
Knowledge Acquisition in Biochemistry, Physiology and Anatomy within the Context of Problem-Based Learning.	Africa Education Review	Hassan, S.	2013	218
Do Creative Thinking Skills in Problem-based Learning Benefit from Scaffolding?	Journal of Turkish Science Education	Ernawati M. D. W. et al.	2023	219
An Investigation of the Pedagogical Impact of Using Case-based Learning in a Undergraduate Biochemistry Course	International Journal of Higher Education	Kulak V., Newtow G.	2015	220
Does the Use of Case-based Learning Impact the Retention of Key Concepts in Undergraduate Biochemistry?	International Journal of Higher Education	Kulak V., Newtow G. Sharma R.	2017	221
Case Based Learning: A Method for Better Understanding of Biochemistry in Medical Students	Journal of Clinical and Diagnostic Research	Nair S. P. et al.	2013	222
Vertical Integration of Biochemistry and Clinical Medicine Using a Near-Peer Learning Model	Biochemistry and Molecular Biology Education	Gallan A. J. Offner G. D. Symes K.	2016	223

The effect of using a case study on the academic achievement of students in learning about the topic of 'Vitamins'	Journal of Biological Education	Günter T Demir F. E. O	2018	224
What is the Effect of Case-Based Learning on the Academic Achievement of Students on the Topic of Biochemical Oxygen Demand?	Research in Science Education	Günter T Alpat S. K.	2019	225
Development and Evaluation of a Multi-Institutional Case Studies-Based Course in Food Safety	Journal of Food Science Education	Pleitner A. M. et al.	2015	226
Online Case-Based Learning for Medical Students as a Teaching Method for Biochemistry at King Abdulaziz University amid COVID-19 Pandemic (a Study Conducted in 2021)	Biochemistry and Molecular Biology Education	Kubbara, E. A. et al.	2024	227
Teaching Protein-Ligand Interactions Using a Case Study on Tau in Alzheimer's Disease	Journal of Chemical Education	Branco, R. C.; Goodson, H. V.; Jonasson, E. M.	2022	228
Assessment of Learning Gains in a Flipped Biochemistry Classroom	Biochemistry and Molecular Biology Education	Ojennus D. D.	2016	229
Flipping a biochemistry class within a medical curriculum: Impacts on perception, engagement, and attainment	Biochemistry and Molecular Biology Education	Fakhoury H. M. A et al.	2021	230
Concept and benefits of the Inverted Classroom method for a competency-based biochemistry course in the pre-clinical stage of a human medicine course of studies	GMS Journal for Medical Education	Kühl S. J. et al.	2017	231
Adopting a flipped classroom approach for teaching molar calculations to biochemistry and genetics students	Biochemistry and Molecular Biology Education	Francis N. et al.	2020	232
Active Learning in Flipped Life Science Courses Promotes Development of Critical Thinking Skills	CBE - Life Sciences Education	Styers M. L. Zandt P. A. V. Hayden K. L.	2018	233
Flipped jigsaw activity as a small group peer-assisted teaching learning tool in Biochemistry Department among Indian Medical Graduate: An experimental study	Biochemistry and Molecular Biology Education	Uppal V. Uppal N.	2020	234
Mixed-mode instruction using active learning in small teams improve generic problem-solving skills of university students	Journal of Further and Higher Education	Klegeris A.	2020	235

Creation and Implementation of a Flipped Jigsaw Activity to Stimulate Interest in Biochemistry among Medical Students	Biochemistry and Molecular Biology Education	Williams C. et al.	2018	236
Increased Preclass Preparation Underlies Student Outcome Improvement in the Flipped Classroom	CBE—Life Sciences Education	Gross et al.	2015	237
Student Comprehension of Biochemistry in a Flipped Classroom Format	Smart Learn. Environ.	Harris, E. N.; Schroder, E. A.; Berks, T. J.	2024	238
Assessment of Blended Biochemistry Classes Based on Massive Open Online Courses and a "Semi-Flipped" Learning Environment	Biochemistry and Molecular Biology Education	Ren, G. et al.	2022	239
Application of Flipped Classroom Combined with Virtual Simulation Platform in Clinical Biochemistry Practical Course	BMC Med Educ	Sun, L. et al.	2023	240
Flipped Classroom versus Traditional Didactic Classroom in Medical Teaching: A Comparative Study	Cureus	Bhavsar, M. H.; Javia, H. N.; Mehta, S. J.	2022	241
Improved Zoom Engagement by Adopting Flipped Class Strategies to the Curriculum of a Biology of Aging Course during the COVID-19 Pandemic	Biochemistry and Molecular Biology Education	Wheaton, K.	2023	242
Flipping Veterinary Biochemistry, Anatomy, and Physiology: Students' Engagement and Perception	Veterinary Sciences	De Brito, C.; Terrado, J.	2024	243
Research on the Application and Effect of Flipped-Classroom Combined with TBL Teaching Model in WeChat-Platform-Based Biochemical Teaching under the Trend of COVID-19	BMC Med Educ	Ji, H. et al.	2023	244
Teaching Graduate Research Skills in Genomics via an Integrated 'Flipped' Journal Club Program	Wiley	Aaron Golden	2022	245
Teaching Metabolism with Scientific Articles: A New Approach	Biochemistry and Molecular Biology Education	Cicuto C. Pazinato M., Torres B.	2018	246
Promoting Active Learning of Graduate Student by Deep Reading in Biochemistry and Microbiology Pharmacy Curriculum	Biochemistry and Molecular Biology Education	Peng R.	2017	247
A Paired Set of Biochemistry Writing Assignments Combining Core Threshold Concepts, Information Literacy, and Real-World Applications	Journal of Chemical Education	Knight J. D. et al.	2021	248

Using The Poisoner's Handbook in Conjunction with Teaching a First- Term General/Organic/Biochemistry Course	Journal of Chemical Education	Zuidema D R. Herndon L. B.	2015	249
A Module Integrating Conventional Teaching and Student-Centered Approach for Critical Reading of Scientific Literature	Biochemistry and Molecular Biology Education	Kulkarni A. Vartak R.	2019	250
Using HeLa Cell Stress Response to Introduce First Year Students to the Scientific Method, Laboratory Techniques, Primary Literature, and Scientific Writing	Biochemistry and Molecular Biology Education	Resendes K. K.	2015	251
Literature-based learning and experimental design model in molecular biology teaching for medical students at Tongji University	Biochemistry and Molecular Biology Education	Wang J. et al.	2020	252
Improving Journal Club: Increasing Student Discussion and Understanding of Primary Literature in Molecular Biology Through the Use of Dialectical Notes	Biochemistry and Molecular Biology Education	McDonough V.	2012	253
Exploration of the scientific papers and self-assessment of students using the COVID-19 case on biochemistry course	Biochemistry and Molecular Biology Education	Anwar Y. A. S. Muti'ah M.	2020	254
Using real-world examples of the COVID-19 pandemic to increase student confidence in their scientific literacy skills	Biochemistry and Molecular Biology Education	Anderson A. E. Justement L. B. Bruns H. A.	2020	255
Diving into Research without Wading through Content: A Skills-Based Cell Biology Course Emphasizing the Unknown	Biochemistry and Molecular Biology Education	González-Del Pino, G. L.; Rokop, M. E.	2024	256
Learning by Teaching Efficiently Enhances Learning Outcomes in Molecular Biology of the Cell Course	Biochemistry and Molecular Biology Education	Mijakovic, I.; Rahimi, S.	2024	257
A Semester-Long Project-Oriented Biochemistry Laboratory Based on Helicobacter pylori Ureasews	Biochemistry and Molecular Biology Education	Farnham K. R. Dube D. H.	2015	258
Exploring the relationship between genotype and phenotype using yeast alcohol dehydrogenase	Biochemistry and Molecular Biology Education	Krzysiak A. Doyle C. Huff M. O.	2022	259
Detection of an ABCA1 Variant Associated with Type 2 Diabetes Mellitus Susceptibility for Biochemistry and Genetic Laboratory Courses	Biochemistry and Molecular Biology Education	Legorreta-Herrera M. et al.	2013	260
Project-based learning in a collaborative group	Journal of Biological Education	Li B. et al.	2019	261

can enhance student skill and ability in the biochemical laboratory: a case study				
Exploring Protein Structure and Dynamics Through a Project-Oriented Biochemistry Laboratory Modulews	Biochemistry and Molecular Biology Education	Lipchock J. M. et al.	2017	262
From Cookbook to Research: Redesigning an Advanced Biochemistry Laboratory	Journal of Chemical Education	Boyd-Kimball B.<br>Miller K. R.	2018	263
A Research-Inspired Biochemistry Laboratory Module–Combining Expression, Purification, Crystallization, Structure-Solving, and Characterization of a Flavodoxin-like Protein	Biochemistry and Molecular Biology Education	Hammerstad M.<br>Røhr Å. K.<br>Hersleth H.	2019	264
Evolution of an 8-week upper-division metagenomics course: Diagramming a learning path from observational to quantitative microbiome analysis	Biochemistry and Molecular Biology Education	Goller C. C.<br>Ott L. E.	2020	265
The Alcohol Dehydrogenase Kinetics Laboratory: Enhanced Data Analysis and Student-Designed Mini-Projects	Journal of Chemical Education	Silverstein T. P.	2016	266
Investigating the Determinants of Substrate Binding through a Semester-Long, Project-Oriented Biochemistry Laboratory Course	Journal of Chemical Education	Sarisky and Johann	2018	267
Introduction of team-based learning improves understanding of glucose metabolism in biochemistry among undergraduate students	Biochemistry and Molecular Biology Education	Eguchi H. et al.	2020	268
Over The Counter Drugs (and Dietary Supplement) Exercise: A Team-Based Introduction to Biochemistry for Health Professional Students	Biochemistry and Molecular Biology Education	Phadtare S<br>Abali E<br>Brodsky B.	2013	269
Verification of learner's differences by team-based learning in biochemistry classes	Korean Journal of medical education	Mun K. H.<br>Mun K. C.	2017	270
Team-based learning versus traditional didactic lectures in teaching clinical biochemistry at King Abdulaziz University; learning outcomes and student satisfaction	Biochemistry and Molecular Biology Education	Alamoudi A. A et al.	2021	271
Mind the Gap: Investigating Disaggregated Student Performance in an Upper-Division Biochemistry Course Following Team-Based Learning	CBE - Life Sciences Education	Woodbury, J.;<br>Offerdahl, E.	2024	272
Adapting Team-Based Learning for an Online Biochemistry Course	Journal of Chemical Education	Woodbury, J.;<br>Arneson, J. B.;<br>Offerdahl, E. G.	2022	273

Is Learning Outcome after Team Based Learning Influenced by Gender and Academic Standing?	Biochemistry and Molecular Biology Education	Das S. at al.	2019	274
Mutual Benefit for Foreign Medical Students and Chinese Postgraduates: A Mixed Team-Based Learning Method Overcomes Communication Problems in Hematology Clerkship	Biochemistry and Molecular Biology Education	Chen X. et al.	2017	275
A Novel Grading Strategy for Team-Based Learning Exercises in a Hands-on Course in Molecular Biology for Senior Undergraduate Underrepresented Students in Medicine Resulted in Stronger Student Performance	Biochemistry and Molecular Biology Education	Carrasco G. A. Behling K. C. Lopez O. J.	2019	276
Effectiveness of Team-Based Learning in teaching Medical Genetics to Medical Undergraduates	Malaysian Journal of Medical Sciences	Ismail N. A. S.	2016	277
Blog Construction as an Effective Tool in Biochemistry Active Learning	Biochemistry and Molecular Biology Education	Rolim E. C. et al.	2017	278
Using Pamphlets To Teach Biochemistry: A Service-Learning Project	Journal of Chemical Education	Harrison M. A. Dunbar V Lopatto D.	2013	279
Case study: Biochemistry without borders: a case study utilising infographics	Essays in Biochemistry	Sahai M. A. Ivanova A.	2022	280
Innovative, integrative, and interactive in-class activity on metabolic regulation: Evaluating educational impacts	Biochemistry and Molecular Biology Education	Marques F. et al.	2021	281
Motivating active learning of biochemistry through artistic representation of scientific concepts	Journal of Biological Education	Bruna C.	2013	282
